# In Vitro Comparison of Differences in Setting Time of Premixed Calcium Silicate-Based Mineral Trioxide Aggregate According to Moisture Content of Gypsum

**DOI:** 10.3390/ma17010035

**Published:** 2023-12-21

**Authors:** Hyun-Jin Kim, Jun-Seok Lee, Dong-Hoon Gwak, Yong-Seok Ko, Chun-Il Lim, Seung-Youl Lee

**Affiliations:** Medical Device Research Division, National Institute of Food and Drug Safety Evaluation, Cheongju 28159, Republic of Korea; khjin0815@korea.kr (H.-J.K.); junseok1025@korea.kr (J.-S.L.); dh0628@korea.kr (D.-H.G.); kys3027@korea.kr (Y.-S.K.); chunil@korea.kr (C.-I.L.)

**Keywords:** premixed calcium silicate MTA, setting time, moisture, gypsum mold, micro-CT

## Abstract

Recently, a paste-type premixed calcium silicate-based mineral trioxide aggregate (MTA) product that quickly solidifies through a pozzolanic reaction was introduced to replace existing MTA, which has the disadvantage of a long setting time. In this study, we evaluated the effect of moisture content in the root canal on the setting time of premixed calcium silicate-based MTA in a simulated root canal environment using Endoseal MTA and Well-Root ST, among commercially available products. The setting time was measured according to ISO 6876/2012. A mold made using grades 2, 3, and 4 dental gypsum according to the classification of ISO 6873/2013 was used to reproduce the difference in moisture environment. Differences in moisture content were measured using micro-computed X-ray tomography (micro-CT). The micro-CT results showed that the moisture content was the highest and lowest in the grade 2 and 4 gypsum molds, respectively. Moreover, the setting time indicated by the manufacturer was the shortest for the grade 2 gypsum mold. Hence, the differences in moisture content significantly affect the setting time of MTA. This result can help set future experimental conditions and develop premixed calcium silicate-based MTA products.

## 1. Introduction

Root canal treatments remove the infected pulp tissue and bacteria present inside a tooth and fill the root canal to induce recovery of the root apex tissue [1]. A successful root canal treatment requires a completely sealed root canal filling to prevent the infiltration of oral bacteria and body fluids into the root canal [2]. Therefore, selecting an appropriate material for filling root canals is critical for thoroughly sealing the apex. An ideal root-end-filling material should be biocompatible, easy to handle, insoluble in body fluids, and safe for pulp [3].

Mineral trioxide aggregate (MTA) is a root-canal-filling material developed by Torabinejad in 1993 [4]. It has advantages such as excellent biocompatibility, bioactivity, low solubility, sealing ability, and antibacterial effects [5] and is widely used in endodontic treatments such as pulp capping, root perforation restoration, and root canal filler in periapical surgery [6]. However, its high price, the challenges in operation, the possibility of discoloration, the content of heavy metals like chrome and challenges in their removal, and its long setting time are the primary disadvantages of MTA [4].

The condensation of MTA is influenced by several factors, such as the mixing ratio and degree, temperature, and humidity [7]. Among these, moisture is the decisive factor affecting the condensation and physical properties of MTA [8]. MTA is a hydraulic cement that reacts with moisture to form a colloidal gel followed by condensation [9]. Excessive moisture may increase the porosity and setting time of the material, causing washing of the MTA and reducing its strength. Conversely, complete condensation may not occur at low mixing ratios or in dry environments, which reduces the strength of the material owing to insufficient hydration [10].

To overcome these shortcomings of traditional MTA, MTA with various materials are being researched and developed [11]. In particular, premixed calcium silicate-based MTA exhibits biocompatibility and osteogenic properties similar to existing MTA. The powder and liquid are not mixed and can be used in a uniform state because the premixed paste-like material is provided in the form of a syringe.

Therefore, MTA can be applied even to narrow and deep root canals, which makes it convenient for users. Additionally, it hardens more quickly by using pozzolan cement with small particles without chemical accelerators [12].

Although this calcium silicate-based MTA is widely used due to its many advantages, performance evaluations according to the clinical environment are not sufficiently available in the relevant literature because the material is provided in a different form compared to existing MTA. Several studies have measured the setting time of MTA using the ISO 6876/2012 (dentistry—root canal sealing materials) [13] method of dental endodontic sealants. However, this approach uses a gypsum mold for materials that require moisture for setting; therefore, accurately evaluating the setting time for moisture sensitive materials like MTA may be challenging.

Therefore, in this study, we simulated the root canal environment using three types of dental gypsum (dental plaster, dental stone, and die stone) with different porosities and compared the effect of moisture content inside the gypsum on the time required for premixed calcium silicate-based MTA to set. The method of the international standard ISO 6876/2012 was followed to measure the setting time, and the moisture content according to the internal structure of each gypsum star was compared using micro-computed X-ray tomography (micro-CT).

## 2. Materials and Methods

### 2.1. Study Material

We selected Endoseal MTA (Maruchi, Wonju-si, Gangwon-do, Korea) and Well-Root ST (Vericom, Chuncheon-si, Gangwon-do, Korea), from among commercially available products with a setting time under 15 min as indicated by the manufacturer, as a premixed calcium silicate-based MTA. The chemical composition of the products and their manufacturers are shown in Table 1.

The gypsum molds used to reproduce the simulated root canal environment were made using snow rock (DK Mungyo, Gimhae-si, Gyeongnam, Republic of Korea) products with grade 2 (dental plaster), grade 3 (dental stone), and grade 4 (die stone) gypsum, according to the classification of ISO 6873/2013 (dentistry—gypsum products) [15]. The mixing ratios and uses are listed in Table 2 according to the type of gypsum indicated by the manufacturer.

### 2.2. Fabrication of Gypsum Molds to Reproduce Simulated Root Canal Environment

**Figure 1:**
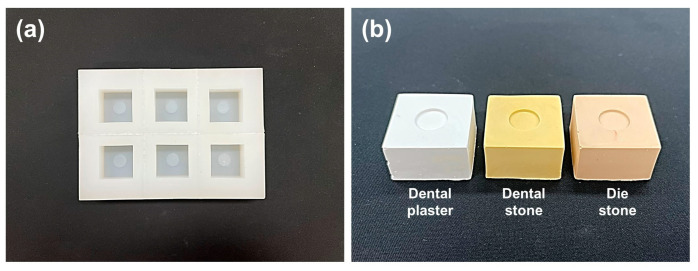
(**a**) Silicone mold for making gypsum casts; (**b**) gypsum cast made using silicone molds. Grade 2: dental plaster; Grade 3: dental stone; Grade 4: die stone.

According to ISO 6876/2012, gypsum molds should be used to evaluate materials that require moisture to set. Therefore, to produce a gypsum mold of the same size that reproduced the simulated root canal environment, a square silicone mold (width 3 cm, height 3 cm, and depth 1.5 cm) with a circular protrusion (diameter 10 mm and thickness 1 mm) was manufactured (Figure 1a).

The prepared gypsums were mixed according to the ratio and method suggested by the manufacturer and slightly overfilled in the manufactured silicone mold. The filled gypsum was covered with a thin polyethylene sheet and glass plate. Once the gypsum mold was completely set, the glass plate and polyethylene sheet were removed and separated from the silicone mold. The irregularities from the surrounding area of the manufactured gypsum mold (Figure 1b) were removed, and it was stored for 24 h in an incubator at 37 °C and a relative humidity of 95%.

### 2.3. Gypsum Surface Measurement Using a Scanning Electron Microscope

A polyethylene sheet was placed on the glass plate. Subsequently, a stainless steel mold with a diameter of 10 mm and a thickness of 1 mm was placed on it. Each gypsum was mixed according to the specified ratio and used to fill in the mold. A polyethylene sheet and glass plate were then placed over the sample in the mold to push out the excess gypsum. After the gypsum specimen was set, it was separated from the mold, and the irregularities around it were carefully removed. The solidified disk-shaped gypsum specimen was coated with platinum (Pt) in an ion-coating device (ion sputter) for 60 s and then mounted on a holder inside a scanning electron microscope. The particle shape of each gypsum surface was observed at a voltage of 5 kV and a magnification of ×2.0 K.

### 2.4. Measurement of Pore Distribution and Moisture Content of Gypsum Using Micro-CT

The moisture distribution according to the internal structure of gypsum was examined in 3D using Micro-CT (Quantum FX; PerkinElmer, Hopkinton, MA, USA). To test this property, a gypsum mold (*n* = 10 for each group) identical to that used to measure the setting time was manufactured and mounted in a graphite sample holder. The X-ray source was set to a voltage of 90 kV and a current of 160 μA, and the CT images were visualized through the 3D viewer software (3D Explore, Ver. 3.0.1; Rigaku Corporation, Akishima-shi, Tokyo, Japan) in the Quantum FX system. The field of view (FOV) was 30 mm, and the filament source–detector distance (SDD) and source–object distance (SOD) were adjusted to obtain a voxel size of approximately 10 μm. After scanning, image processing was performed using AVIEW software (AVIEW Modeler version, 1.0.3; Coreline Software, Seoul, Republic of Korea). To maximize the inclusion of all the pores in the gypsum, the volume of interest (VOI, 21 mm × 12.5 mm) was set as close to the boundary of the specimen as possible, excluding the cavity portion of the gypsum mold. The noise was removed from the selected image using a median filter; the intensity of the image was segmented to display the objects of interest (pores and moisture inside the gypsum), and the small unimportant parts were removed. The distribution of the pores was analyzed by comparing the surface area of each pore inside the gypsum, and the moisture content was measured as the pore volume relative to the total volume of the gypsum.

### 2.5. Setting Time Measurement

The tip was attached to an injection containing the premixed calcium silicate-based MTA. The MTA samples were filled into the cavity (diameter: 10 mm; height: 1 mm) of a gypsum mold (Figure 1b) stored at a constant temperature in a humidity chamber for 24 h. Then, they were placed on a metal block at a constant temperature of 37 °C in a humidity chamber with 95% relative humidity. Five minutes after the sample was mixed, a Gilmore needle (weight 100 g and diameter 2 mm) was carefully lowered vertically onto its surface to check whether indentations had formed. The tip of the Gilmore needle in contact with the specimen was wiped clean each time, and the needle was lowered to a new location on the specimen. The process of lowering the Gilmore needle was repeated until no indentations were formed, and the time from the end of mixing until no indentations were observed was recorded as the setting time. The same test was repeated 10 times to obtain average values and standard deviations.

### 2.6. Statistical Analysis

The Kolmogorov–Smirnov test was performed to test normality, and the results confirmed that it followed a normal distribution. The moisture content of each gypsum and the setting time measurement results of MTA were statistically tested by post hoc analysis using the Kruskal–Wallis rank sum test and the Mann–Whitney U test. All statistical significance levels were set at 0.05 (5%), and the statistical analysis was performed using predictive analysis software (IBM SPSS Statistics 25.0; IBM, Armonk, NY, USA).

## 3. Results

### 3.1. Surface Particle Morphology Analysis of Gypsum

**Figure 2:**
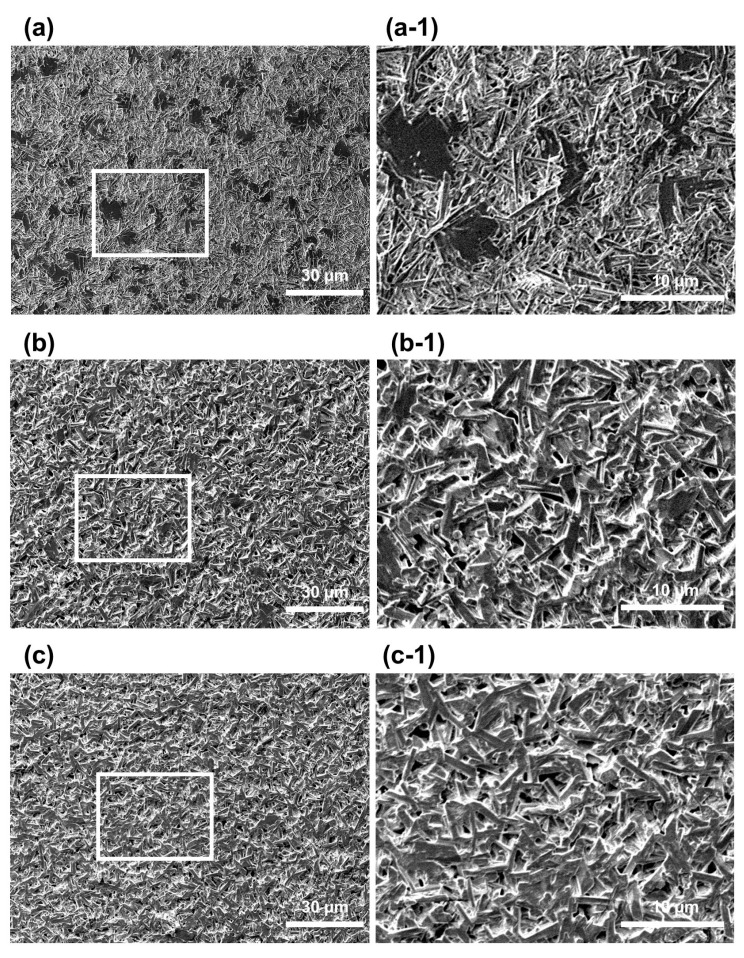
Scanning electron micrograph showing particle size and shape of the set gypsum surface: (**a**,**a-1**) dental plaster; (**b**,**b-1**) dental stone; (**c**,**c-1**) die stone. Magnification: (**a**–**c**) 500× *g*; (**a-1**–**c-1**) 3000× *g*.

Figure 2 shows the shapes of the particles on the surface of the gypsum specimen measured using a scanning electron microscope. The shape of the surface particles of each gypsum was such that the grade 2 gypsum (Figure 2(a,a-1)) had irregularly shaped, elongated particles dispersed throughout the surface, and large and small pores were present in areas where the particles were not aggregated. In contrast, the surface of grade 3 gypsum had aggregated particles of more uniform size than those of grade 3 gypsum (Figure 2(b,b-1)), and the spacing between particles was narrow; thus, there were fewer pores that were smaller and more widely distributed. The particles were the most densely aggregated in grade 4 gypsum (Figure 2(c,c-1)), and the spacing between particles was the narrowest; thus, pores hardly appeared.

### 3.2. Analysis of Pore Distribution and Moisture Content of Gypsum Using 3D Images of Micro-CT

**Figure 3:**
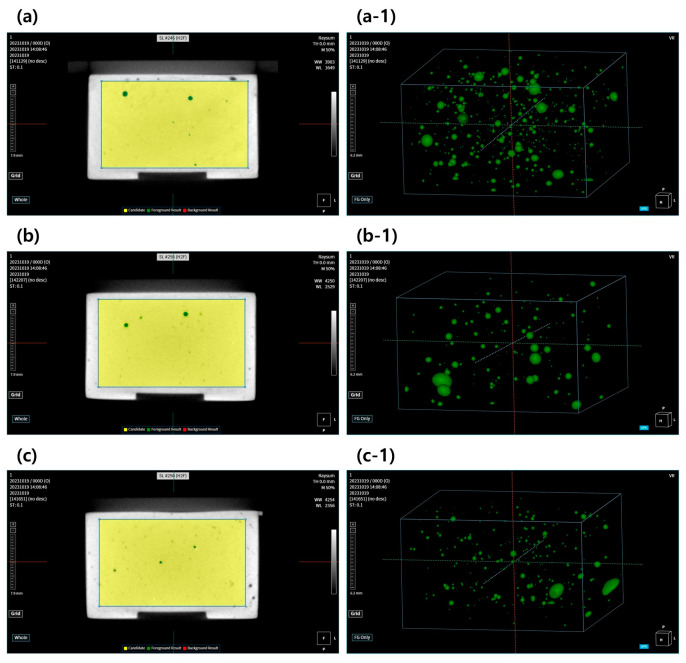
VOI of gypsum mold and volume rendering of the pore distribution (green) within the segments of each gypsum mold obtained through micro-CT analysis. (**a**,**a-1**) Dental plaster; (**b**,**b-1**) dental stone; (**c**,**c-1**) die stone.

**Figure 4:**
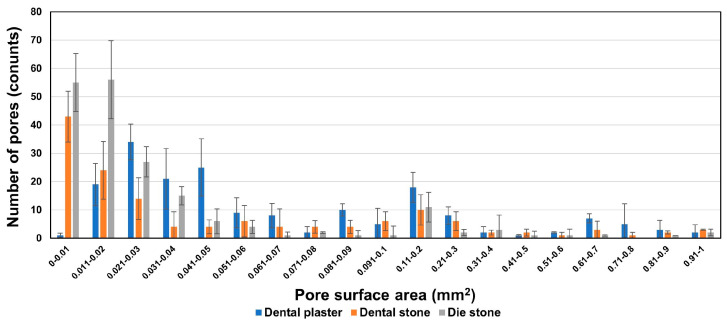
Surface area and number distribution graph of gypsum pores measured by micro-CT.

Figure 3 shows a 3D image of internal structure in the gypsum mold obtained using micro-CT, and the pore distribution is shown in Figure 4. Grade 2 gypsum (Figure 3(a-1)) had large and small pores widely distributed throughout the material, and the overall surface area of the pores was the largest (Table 3). Additionally, the number of pores with a large surface area was greater than that of other gypsums (Figure 4). Grade 3 gypsum (Figure 3(b-1)) also had a mixture of large and small pores, but the total surface area of pores was lower than that of grade 2 gypsum (Table 3), and the distribution of pores showed that there were more small pores than large pores (Figure 4). The pores inside Grade 4 gypsum (Figure 3(c-1)) were distributed throughout, but there were more small pores than large pores, so the surface area was the smallest (Figure 4).

The moisture content according to the pore distribution of each grade of gypsum is listed in Table 3. The moisture content was expressed as a percentage (%) of the pore volume relative to the total volume of gypsum, and the moisture content of grade 2 gypsum, which had the largest number of pores with a large surface area, was the highest at 1.02%. Because the moisture content of grade 4 gypsum was the lowest at 0.41% (Table 3), the moisture content inside the gypsum mold was classified as grade 2 (dental plaster) > grade 3 (dental stone) > grade 4 (die stone) depending on the volume of internal pores, decreasing in that order. Therefore, the pore surface area and moisture content of grade 2 gypsum are significantly different from those of grade 4 gypsum (*p* < 0.05).

### 3.3. Measurement of Setting Time According to the Moisture Content of the Gypsum Mold

The setting times of Endoseal MTA were 5.8 min, 7.5 min, and 15.6 min in grade 2, 3, and 4 gypsum, respectively, with the shortest setting time reported in grade 2 gypsum with high pore distribution and moisture content. The setting times of Well-Root ST were reported as 10.4 min, 17.5 min, and 23.9 min for grade 2, 3, and 4 gypsum, respectively (Table 4). Therefore, for both products, the shortest setting time was observed for grade 2 gypsum and the longest time was observed for grade 4 gypsum. Therefore, the setting time of premixed calcium silicate-based MTA using grade 2 gypsum is significantly different from the setting time using grade 4 gypsum (*p* < 0.05).

## 4. Discussion

MTA is a hydraulic cement developed based on Portland cement [16]. It hardens as the powder reacts with moisture to form a colloidal gel [9,17]. This cement is influenced by temperature, pressure, humidity, the type of material, etc. However, it is most affected by the powder-to-liquid ratio [18,19,20]. In particular, the existing MTA is primarily used by mixing powders and liquids and has the disadvantage of being sensitive to the mixing ratio and user skill level, which makes reproducing uniform results somewhat challenging [21]. Manufacturers recommend a water/powder ratio of approximately 1:3 to form a uniform paste of MTA [22,23]. The literature also suggests a water/powder ratio of 0.3 to 0.6 to obtain an acceptable concentration that increases as hydration progresses [24].

However, unlike existing MTA, which has a fixed powder-to-liquid ratio, the amount of moisture required for hardening is not separately specified for calcium silicate-based MTA that comes premixed from the manufacturer. Moreover, the manufacturer only indicates that hardening was achieved by the moisture within the root canal [25,26].

To evaluate the setting time of the premixed calcium silicate-based MTA, we considered existing methods to evaluate the performance of MTA. However, there are no international standards or specifications for testing MTA, and tests related to MTA are being evaluated in various studies and clinical trials [8].

Measuring the setting time using an indentation needle in ISO 9917-1 [27] and ISO 6876 is the most commonly used method to evaluate the performance MTA [28,29]. However, because MTA does not solidify unless mixed with water, the method of measuring the setting time according to ISO 9917-1 by evaluating the physical performance of restorative cements that solidify in a dry environment is not appropriate [8]. Conversely, ISO 6876 is a standard that was designed to test sealer cements used to help close root canals. In this standard, the amount of moisture required for hardening is specified accurately, or stainless steel molds are used for materials that harden in dry conditions. In contrast, for hydraulic cement such as MTA that requires moisture to harden, setting time is measured using a gypsum mold [13]. Koo et al. [29] used ISO 6876 stainless steel molds and gypsum molds to compare the setting times of calcium silicate-based bio-ceramic sealers and reported that the gypsum molds showed a significantly shorter setting time as a result. Following these previous studies, we also used a gypsum mold to measure the setting time of premixed calcium silicate-based MTA. However, because the shape of the root canal and the amount of water contained within the dentinal tubules differ for each root canal of the tooth, the same amount of water is not always applied to the setting of the MTA.

Therefore, in this experiment, three types of dental gypsums with different porosities were used to reproduce the diversity within the simulated root canal and to determine the effect of differences in moisture inside the root canal on the setting time of the premixed calcium silicate MTA.

The dental gypsums (Table 2) used in this study were grade 2 (dental plaster), grade 3 (dental stone), and grade 4 (die stone), which are used to create models and impressions in dentistry. Dental gypsum differs in density and internal structure depending on the shape, size, and type of the particles.

Gypsum is divided into plaster, stone, and high-strength dental stone according to the method of removing moisture from the material. Plaster has a heterogeneous hardening state and many air bubbles, but the hardened structure of stone and high-strength dental stone is homogeneous and dense [30].

The pores in dental gypsum occur when air is trapped in the material during the mixing process, and pores form between crystals when the crystals of gypsum powder grow [31]. The pores of this dental gypsum can be identified as dark areas in scanning electron microscope images (Figure 2) [32]. In this experiment, the plaster, which was a grade 2 gypsum, showed large spaces created by pores as dark areas during mixing, and some pores appeared as dark areas between the particles (Figure 2(a-1)). Grade 2 and 3 gypsum showed slightly more dense crystal growth, and almost no large pores were visible on the surface. It also showed a tissue type in which only small pores were present between crystals (Figure 2(b-1,c-1)). As a result, this study also confirmed that when mixing gypsum powder, pores appear inside the gypsum and between gypsum crystals. Therefore, it was found that there were differences in the internal structure of the hardened gypsum because the size and shape of the particles were different depending on the type of gypsum.

Although an analysis using scanning electron microscopy provides high-resolution images at the nanometer scale, it has the disadvantage of requiring cross-sectioning of the sample, and the two-dimensional (2D) image does not provide spatial information about the structure. On the other hand, micro-CT produces three-dimensional (3D) images with high resolution and can be used to check volume, shape, space, and size distribution [33]. It also has the advantage of not destroying the internal structure and materials of the tissue [34]. For this reason, micro-CT has recently been widely adopted in dentistry and other fields to evaluate the porosity of materials and to analyze pores. Neves et al. [35] compared and evaluated the porosity and pore size of glass ionomer cements with different viscosities using micro-CT, and Guerrero at al. [36] compared the porosity of Fillapex and BioRoot MTA for endodontic treatment. Similarly, Rattanassak et al. [37] investigated the pore structure characteristics of cement/pozzolan composites using micro-CT.

A CT analysis generates images based on density differences inside an object so that the presence of pores or water can be confirmed or the degree of distribution can be observed. Areas where water is present in the gypsum can be distinguished from the surrounding tissue owing to differences in density, as shown in Figure 3, and the presence of water can be confirmed more clearly by appropriately setting the area of interest and scanning conditions (Figure 3(a-1–c-1)) [38].

In contrast, when the areas of gypsum and moisture were removed from the image, the areas of pores could not be identified. However, the pores inside the gypsum provide a space to absorb water molecules from the surrounding environment or to store the absorbed water [39], so we considered that all empty spaces were pores and that all spaces were filled with moisture. Therefore, the moisture content was expressed as the total volume (Table 3) of the green parts in the 3D image, and as a result, the moisture content showed significant differences depending on the type of gypsum.

However, the analysis program used in this experiment only provided the volume and surface area of the portion selected as pores, and the pores inside the gypsum were not only circular but also irregular in shape, so their exact size could not be classified. Therefore, any area where moisture was present was judged to be a pore, and the tendency of pore distribution was determined based on the surface area of each pore. These results are a limitation of this study, and additional research using analysis instruments or programs with higher resolution and sensitivity is needed to analyze pore size [40].

In this study, we compared differences in setting time by applying different amounts of moisture that can react with the widely used premixed calcium silicate MTA. One key limitation of this study is that the differences in the setting time of MTA alone were observed depending on gypsum with different moisture contents; therefore, additional research is needed to determine the effect of moisture content on its mechanical and physical properties. Additionally, because the performance of MTA is affected by the conditions used in the testing environment, serious concerns may arise if the standardized tests are not representative of the clinical environment. We evaluated the setting time of the premixed calcium silicate-based MTA by referring to the test method presented in ISO 6876. The pore distribution and moisture content of gypsum differed depending on the type, and the setting time of MTA also showed differences. Therefore, moisture was required to evaluate the setting time of the premixed calcium silicate-based MTA, and the results showed that if the moisture was insufficient, the setting time could be delayed significantly.

In conclusion, the setting time of premixed calcium silicate-based MTA varies depending on the moisture content inside the gypsum. In addition, the setting time was the shortest when using the grade 2 gypsum mold presented in the guidelines of ISO 6876/2012.

## 5. Conclusions

This study evaluated the setting time of premixed calcium silicate-based MTA according to ISO 6876 and compared it by injecting the MTA into three grades of gypsum molds with different moisture contents. The setting time of the MTA was the fastest when using a gypsum mold made of highly porous grade 2 gypsum.

Therefore, it was confirmed that the difference in moisture content could significantly affect the setting time of MTA. This information can be useful for selecting future experimental conditions and developing premixed calcium silicate-based MTA products.

## Figures and Tables

**Table 1:** Chemical composition of the commercial premixed calcium silicate-based MTA used in this study [14].

Materials	Composition	Manufacture
Endoseal MTA	Calcium silicates (dicalcium silicate), tricalcium aluminates, calcium aluminoferrite, calcium sulfates, bismuth oxide, zirconium oxide, thickening agents	Maruchi, Wonju, Republic of Korea
Well-Root ST	Calcium silicate compound, calcium sulfate dehydrate, calcium sodium phosphosilicate, zirconium oxide, titanium oxide, thickening agents	Vericom, Chuncheon, Republic of Korea

**Table 2:** Classification of commercial dental gypsums used in this study.

Materials	Type	Water/Powder Ratio	Use
Dental plaster	2	45 mL/100 g	Study and mounting use
	Hard		
Dental stone	3	23 mL/100 g	Various purposes
	Very hard		
Die stone	4	20 mL/100 g	High strength and low expansion, detailed work
	Ultra hard		

**Table 3:** Pore volume, surface area, and moisture content inside the gypsum measured by micro-CT.

Gypsum Product	Whole Volume (mm^3^)	Pore Volume (mm^3^)	Pore Surface Area (mm^2^)	Moisture Content (%)
Dental plaster	1862.6 ± 6.04	19.1 ± 1.47 ^a^	162.54 ± 4.69 ^a^	1.02 ± 0.07 ^a^
Dental stone	1865.7 ± 4.91	12.2 ± 0.91 ^b^	91.1 ± 3.27 ^b^	0.65 ± 0.04 ^b^
Die stone	1863.5 ± 6.53	7.8 ± 0.70 ^c^	73.64 ± 3.97 ^c^	0.41 ± 0.03 ^c^

Different lowercase superscripts indicate statistically significant differences between type of gypsums (*p* < 0.05).

**Table 4:** Setting time of premixed calcium silicate-based MTA according to gypsum type.

Type of Molds	Setting Time (min)	
	Endoseal MTA	Well-Root ST
Dental plaster	5.8 ± 0.30 ^a^	10.4 ± 0.41 ^a^
Dental stone	7.5 ± 0.37 ^b^	17.5 ± 0.38 ^b^
Die stone	15.6 ± 0.51 ^c^	23.9 ± 0.78 ^c^

Different lowercase superscripts indicate statistically significant differences between type of gypsums (*p* < 0.05).

## Data Availability

Data are contained within the article.
